# Supplementary material for: Verbal and psychological violence against women in Turkey and its determinants
Source: PLoS One. 2022 Oct 10;17(10):e0275950. doi: 10.1371/journal.pone.0275950 (PMC9550074; doi:10.1371/journal.pone.0275950)
Supplement: S1 Appendix — (PDF) [file pone.0275950.s001.pdf]

Appendix 1. Estimated binary probit regression model results and marginal effects related to factors affecting women's exposure to verbal and psychological violence

Variables	β	Std. Error	95% CI Lower	95% CI Upper	Elasticity (%)	Std. Error
**Survey year (reference: 2008)**						
2014	0.083[a]	0.027	0.029	0.136	8.23[a]	0.027
**Region (reference: west)**						
South	-0.011	0.043	-0.095	0.072	-1.16	0.043
Middle	0.063[c]	0.035	-0.006	0.131	6.23[c]	0.035
South	-0.066	0.042	-0.148	0.015	-6.83	0.043
East	0.047	0.037	-0.026	0.120	4.71	0.037
**Place of residence (reference: rural)**						
Urban	-0.01	0.03	-0.064	0.050	-0.69	0.029
**Age (reference: 55+)**						
15-24	0.019	0.072	-0.122	0.161	2.02	0.075
25-34	0.112[c]	0.057	0.000	0.225	11.35[c]	0.059
35-44	0.057	0.056	-0.053	0.166	5.81	0.058
45-54	0.060	0.056	-0.049	0.169	6.18	0.058
**Educational level (reference: illiterate)**						
Elementary school	0.079[c]	0.043	-0.006	0.164	8.21[c]	0.046
Secondary school	0.096	0.062	-0.025	0.216	9.90	0.059
High school	0.154[b]	0.060	0.037	0.270	15.60[b]	0.058
University	0.171[b]	0.074	0.026	0.316	17.30[b]	0.074
**Individual earning/income (reference: no)**						
Yes	-0.017	0.035	-0.085	0.051	-1.68	0.035
**Health insurance status (reference: no)**						
Yes	-0.083[b]	0.039	-0.160	-0.006	-8.18[b]	0.038
**Marital status (reference: two and more)**						
Never married	-0.272[b]	0.108	-0.485	-0.060	-24.46[b]	0.097
Once	-0.318[a]	0.080	-0.475	-0.160	-28.99[a]	0.066
**Health status (reference: excellent/good)**						
Reasonable	0.147[a]	0.029	0.090	0.205	14.91[a]	0.030
Bad/very bad	0.233[a]	0.046	0.142	0.323	22.90[a]	0.044
**Number of children (reference: has no child)**						
One child	0.090	0.060	-0.028	0.207	9.43	0.064
Two and more	0.160[a]	0.056	0.050	0.271	16.51[a]	0.060
**Exposure to violence by first-degree relatives (reference: no)**						
Yes	0.334[a]	0.045	0.246	0.423	30.78[a]	0.038
**Husband/partner's educational level (reference: elementary school)**						
Illiterate	-0.048	0.073	-0.192	0.095	-5.03	0.077
Secondary school	0.099[b]	0.042	0.018	0.181	9.87[b]	0.041
High school	0.092[b]	0.038	0.017	0.166	9.13[b]	0.038
University	-0.027	0.052	-0.129	0.075	-2.79	0.054
**Husband or partner's employment status (reference: no)**						
Yes	-0.007	0.036	-0.078	0.064	-0.70	0.036
**Husband or partner's alcohol use status (reference: no)**						
Yes	0.216[a]	0.034	0.148	0.283	20.79[a]	0.032
**Husband or partner's gambling status (reference: no)**						
Yes	0.279[a]	0.099	0.084	0.473	25.61[a]	0.083
**Husband or partner's drug use status (reference: no)**						
Yes	0.316	0.249	-0.171	0.804	28.69	0.202
**Husband or partner's cheating status (reference: no)**						
Yes	0.490[a]	0.054	0.384	0.595	42.65[a]	0.040
**Status of exposure to husband or partner's economic violence (reference: no)**						

Yes	0.367[a]	0.032	0.305	0.429	34.56[a]	0.028
**Status of exposure to husband or partner's physical violence (reference: no)**						
Yes	1.148[a]	0.030	1.089	1.207	97.30[a]	0.024
**Status of exposure to husband or partner's sexual violence (reference: no)**						
Yes	0.772[a]	0.049	0.676	0.868	62.23[a]	0.031
**Constant**	-0.991[a]	0.119	-1.226	-0.758		

[a]p<.01; [b]p<.05; [c]p<.10
