# Supplementary material for: Verbal and psychological violence against women in Turkey and its determinants
Source: PLoS One. 2022 Oct 10;17(10):e0275950. doi: 10.1371/journal.pone.0275950 (PMC9550074; doi:10.1371/journal.pone.0275950)
Supplement: Attachment — Submitted filename: Response to reviewers-05.07.2022.docx [file pone.0275950.s002.docx]

**Editor’s Evaluation**

Thank you for submitting your manuscript to PLOS ONE. After careful consideration, we feel that it has merit but does not fully meet PLOS ONE’s publication criteria as it currently stands. Therefore, we invite you to submit a revised version of the manuscript that addresses the points raised during the review process.

Your manuscript has been assessed by an expert reviewer, whose comments are appended below. As you will see, the reviewer raises several concerns regarding the contextualisation of the research within the existing literature, aspects of the methodology, and framing of the results and conclusions. Please ensure you respond to all of these points in your response to reviewers, and revise your manuscript accordingly.

Please submit your revised manuscript by Jun 24 2022 11:59PM. If you will need more time than this to complete your revisions, please reply to this message or contact the journal office at plosone@plos.org. When you're ready to submit your revision, log on to https://www.editorialmanager.com/pone/ and select the 'Submissions Needing Revision' folder to locate your manuscript file.

Please include the following items when submitting your revised manuscript:

A rebuttal letter that responds to each point raised by the academic editor and reviewer(s). You should upload this letter as a separate file labeled 'Response to Reviewers'.

A marked-up copy of your manuscript that highlights changes made to the original version. You should upload this as a separate file labeled 'Revised Manuscript with Track Changes'.

An unmarked version of your revised paper without tracked changes. You should upload this as a separate file labeled 'Manuscript'.

**AUTHORS’ RESPONSE**

Thank you for the opportunity to revise our paper. We definitely took the feedback of the reviewers to heart and incorporated the suggested revisions.

**Reviewers' comments:**

**Reviewer #1:** This article addresses an important topic, i.e., psychological violence against women, which has received less attention than other forms of violence. Specifically, the authors use secondary data to assess correlates of psychological violence in Turkey. Despite the relevance of the topic, there are a number of issues that limit the contribution of the article to the literature. I identify some of them below and provide recommendations on how to address them.

**Comment:** Thank you for the comment. We definitely took the feedback of yours to heart and incorporated the suggested revisions.

1) Literature review: In my view, this is one the weakest sections of the article. The review is quite general and does not offer a synthesis of previous research (e.g., what do we know about the prevalence of psychological violence in Turkey and other countries, how does it compare to other forms of violence, what factors are associated with it, what are the current gaps in the literature, etc.). Because of this, the contribution of the article is unclear, as is the selection of correlates to be included in the models, which seems to be driven more by availability than theory or previous research findings. I would encourage the authors to revisit this first section to make it more specific and link it to their analytical decisions (e.g., by justifying the inclusion of variables, and stating specific hypotheses to test).

**Comment:** Thank you for the comment. The introduction and literature review of the study was re-written in detail as per the comments of the Reviewer, explaining how the study contributes to the current literature. The Turkey literature of the study was rewritten in detail.

In addition to this, some assumptions are made, with statements not being backed by appropriate citations (e.g., first sentence “It is observed that violent acts are becoming increasingly widespread in today's social life”).

The following book chapter, devoted to the topic of psychological violence against women, might be helpful to incorporate in your review: Aizpurua & O´Connell (2020). Men's psychological violence against women. The SAGE Handbook of Domestic Violence. Sage.

**Comment:** Thank you for the comment. The studies recommended by the Reviewer were reviewed in detail and the introduction and literature review section was rewritten to be used in the paper.

2) Methods: I understand that the authors are using secondary data, but much information is missing from this section to properly assess the methodology of the study. At a minimum, the article should include: response rates (given their potential impact on non-response error), language(s) in which the survey was administered, data collection dates, survey mode, and any incentives offered to participants. Because two rounds/waves of data are used, information on the consistency of data collection procedures and instruments is essential to ensure that the data from 2008 and 2014 are comparable.

**Comment:** Thank you for the comment. The methods section of the study was re-written in detail as per the comments of the Reviewer. The required explanations about the sample and data were added to the method section in accordance with the criticism of the reviewer.

In addition to this, the authors should state how was missing data handled (e.g., multiple imputation, complete case analysis, in which case, it would be helpful to provide evidence that data was missing at random).

**Comment:** Thank you for the comment. The required explanations about the sample and data were added to the method section in accordance with the criticism of the reviewer.

Although the operationalisation of most variables is described in this section, I don´t think the authors explain how were other forms of violence measured, and what the timeframes were. Specifying the timeframe used in the question wording is also relevant for the main outcome (psychological violence), as it will influence estimates and the ability to compare estimates from this study with prior research.

**Comment:** Thank you for the comment. The required correction has been performed in accordance with the criticism of the reviewer. Considering the comments of the Reviewer, “…status of exposure to husband/partner's economic violence at any point in her life (no, yes), status of exposure to husband/partner's physical violence at any point in her life (no, yes), and status of exposure to husband/partner's sexual violence at any point in her life (no, yes).” statements have been incorporated into the article under the “Measures and variables” section.

I do appreciate the authors using weights in their analysis, but I encourage them to specify what type of weights were used for transparency and replicability.

**Comment:** Thank you for the comment. The required correction has been performed in accordance with the criticism of the reviewer. Considering the comments of the Reviewer, “The computed weights of women were added to these data sets in accordance with the with the sample design of the research. Each cluster was assigned a different weight; the reasons for this can be summarized as follows: 1) Differential selection probabilities at the cluster level; 2) Non-proportional distribution of the sample size; and 3) Differential response rates in each stratum” statements have been incorporated into the article under the “Study size” section.

3) Results: Given the consistency of logit and probit models, it is unclear to me why the authors decided to report both. I wonder if presenting one of them and having the other models in the Appendix -and a note regarding consistency in the main text- would help streamline the findings, as the added value of having both sets of analysis in the main text is unclear at the moment.

Thank you for the comment. The result section of the study was re-written in detail as per the comments of the Reviewer.

I also have questions regarding the model strategy, and the decision to estimate one model with the women´s variables and another model with the husbands´ variables. I would, instead, suggest estimating nested models (the first one including only women-related variables, and then the full model). This way, you control for all variables in the full model and can see how model specification influences the findings. There is one article that examined correlates of psychological violence against women in Spain using secondary data which included both sets of variables, which might be helpful: Aizpurua et al. (2018). Controlling Behaviors and Intimate Partner Violence Among Women in Spain: An Examination of Individual, Partner, and Relationship Risk Factors for Physical and Psychological Abuse. Journal of interpersonal Violence. <https://doi.org/10.1177/0886260517723744>).

**Comment:** Thank you for the comment. The studies recommended by the Reviewer were reviewed in detail. We actually estimated only one model. However, we have shown the estimation results in two separate tables. This was not appropriate. The required correction has been performed in accordance with the criticism of the reviewer. We have given all the estimation results in a single table.

A more minor question has to do with the selection of reference categories in your models. It would be helpful to choose a consistent criterion, such as the most frequent category.

**Comment:** Thank you for the comment. Considering the comments of the Reviewer, “The problem of multicollinearity in the models was taken into account while identifying the reference category for ordinal and nominal variables having more than two categories. In this regard, the best model was tried to be estimated. Therefore, a consistent criterion could not be selected” statements have been incorporated into the article under the “Measures and variables”.

4) Discussion: Although you acknowledge the limitations of the cross-sectional research design, sometimes causal language is used, which is not appropriate. I would encourage the authors to review the article to ensure that those references are removed. I would also suggest further discussing some limitations of the research, such as the few indicators used to measure psychological violence, or the exclusion of important age groups from the population (women 60 and over). More broadly, I think this section could be streamlined by providing less details about individual studies and situating the findings within the literature a bit more broadly (what is consistent, what is inconsistent), as well as linking the finding with theory, and not only empirical findings.

**Comment:** Thank you for the comment. The discussion section was reorganized in accordance with the criticism of the Reviewer. Considering the comments of the Reviewer, “Finally, the data in the study consists of women between the ages of 15-59. Since a sample will be created across Turkey, women aged 60 and over were excluded from the study because the likelihood of women aged 15-59 in the visited houses was higher” statements have been incorporated into the article under the “Conclusions” section.

I hope these comments are helpful and wish the authors the best as they move forward with this manuscript.

**Comment:** Thank you for the comment. We definitely took the feedback of yours to heart and incorporated the suggested revisions.
