# Supplementary material for: Verbal and psychological violence against women in Turkey and its determinants
Source: PLoS One. 2022 Oct 10;17(10):e0275950. doi: 10.1371/journal.pone.0275950 (PMC9550074; doi:10.1371/journal.pone.0275950)
Supplement: Attachment — Submitted filename: Response to Reviewers-19.09.2022.docx [file pone.0275950.s003.docx]

**Editor’s Evaluation,**

Thank you for submitting your manuscript to PLOS ONE. After careful consideration, we feel that it has merit but does not fully meet PLOS ONE’s publication criteria as it currently stands. Therefore, we invite you to submit a revised version of the manuscript that addresses the points raised during the review process.

**AUTHORS’ RESPONSE**

Thank you for the opportunity to revise our paper. We definitely took the feedback of the academic editor to heart and incorporated the suggested revisions.

**ACADEMIC EDITOR:**

**Comment:** Literature review: It is usually not expected to have a section designated as literature review in the introduction of a manuscript. However, a review of the literature would be done in the introduction section. I therefore suggest that the authors remove the subtitle "Literature review" from the introduction and further summarise the section. It is quite long.

**Response:** Thank you for the comment. We removed the subtitle "Literature review" from the introduction. The relevant section has been shortened.

**Comment:** It is important for an English language editor to review and revise the manuscript.

**Response:** Thank you for the comment. Language and grammatical errors were corrected during the “proofreading” made by the language editor of Scribendi.

**Comment:** Methodology: It should be stated that this present study was a secondary date analysis

**Response:** Thank you for the comment. Taking this criticism into account, we added relevant expression in the subtitle Data sources/measurement.

**Comment:** Statistical analysis: There are some data analysis that were done and reported in the result section but was not described in the statistical analysis section. e.g VIF. Authors should describe all the statistical analysis conducted. Please explain how the models were built.

**Response:** Thank you for the comment. Necessary amendments were made following the comments of the academic editor.

**Comment:** Results:

Line 411: delete "chi-square tests" and replace with "bivariate analysis"

Table one . change p-value = 0.0000 to p-value < 0.0001

Line 423: change "ratio" to "prevalence"

Line 426: 78.6 should be 78.6%

Line 426: Delete "great"

Line 426: please delete "great"

**Response:** Thank you for the comment. Necessary amendments were made following the comments of the academic editor.

**Comment:** Table 2: please report odds ratio instead of beta. odds ratio is easily explained for logistic regression than beta. Then please interprete appropriately based on the odds ratio.

**Response:** Thank you for the comment. We revised Table 2. and reported odds ratio instead of beta.

**Comment:** Conclusion is too long. Please shorten it to the Essentials

**Response:** Thank you for the comment. The relevant section has been shortened. We added the subtitles Limitations of the study and Directions/suggestions for future research.
