# Supplementary material for: Verbal and psychological violence against women in Turkey and its determinants
Source: PLoS One. 2022 Oct 10;17(10):e0275950. doi: 10.1371/journal.pone.0275950 (PMC9550074; doi:10.1371/journal.pone.0275950)
Supplement: Attachment — Submitted filename: Response to Reviewers-23.09.2022.docx [file pone.0275950.s004.docx]

**Additional Editor Comments:**

**Comment:** Introduction: Please reduce the length of the introduction. Introduction beyond 1000 words suggests a long introduction.

**Response:** Thank you for the comment. The introduction section has been shortened. It was shortened from 2185 words to 1045 words.

**Comment:** Italic: Please avoid italics as much as possible. You have so many italics in the manuscript

**Response:** Thank you for the comment. Necessary amendments were made following the the comments of the academic editor.

**Comment:** Interpretation of odds ratio. Authors should consult an experienced statistician to guide in the interpretation of odds ratio. Current odds ratio interpretations are not correct.

**Response:** Thank you for the comment. We consulted an experienced statistician to guide in the interpretation of odds ratio. Necessary amendments were made following the comments of the academic editor.
